# Variation in brief treatment for substance use disorder: a qualitative investigation of four federally qualified health centers with SBIRT services

**DOI:** 10.1186/s13011-021-00381-y

**Published:** 2021-07-14

**Authors:** Dennis P. Watson, Monte D. Staton, Michael L. Dennis, Christine E. Grella, Christy K. Scott

**Affiliations:** 1 Chestnut Health Systems, 221 W Walton St, 60610 Chicago, IL United States; 2 Center for Dissemination and Implementation Science, Department of Medicine, College of Medicine, University of Illinois at Chicago, 818 S. Wolcott, 60612 Chicago, IL United States; 3 Chestnut Health Systems, 448 Wylie Dr, 61761 Normal, IL United States

**Keywords:** Brief treatment, Screening, brief intervention, and referral to treatment, SBIRT, Substance use disorder, Alcohol use disorder, Outpatient treatment, Recovery management checkups

## Abstract

**Background:**

Brief treatment (BT) can be an effective, short-term, and low-cost treatment option for many people who misuse alcohol and drugs. However, inconsistent implementation is suggested to result in BT that often looks and potentially costs similar to regular outpatient care. Prior research is also rife with inconsistent operationalizations regarding the measurement of BT received by patients. As such, there is a need to more explicitly identify and document variations in BT practice.

**Methods:**

A qualitative investigation of BT in four Federally Qualified Health Centers (FQHC) was undertaken as a sub study of a larger clinical trial. Researchers interviewed 12 staff (administrators and clinicians) involved in BT oversight, referral, or delivery within the four FQHCs. Data were analyzed following an inductive approach guided by the primary research questions.

**Results:**

Findings demonstrate considerable differences in how BT was conceptualized and implemented within the FQHCs. This included a variety of ways in which BT was presented and described to patients that likely impacts how they perceive the BT they receive, including potentially not understanding they received substance use disorder treatment at all.

**Conclusions:**

The findings raise questions regarding the validity of prior research, demonstrating more objective definitions of BT and fidelity checklists are needed to ensure integrity of results. Future work in this area should seek to understand BT as practiced among a larger sample of providers and the direct experiences and perspectives of patients. There is also a need for more consistent implementation, quality assurance guidelines, and standardized stage of change assessments to aid practitioners.

## Background

Referral to brief treatment (BT) or regular outpatient alcohol use or substance use disorder (SUD) treatment is a key component of the Screening, Brief Intervention, and Referral to Treatment (SBIRT) model [1, 2], which is a widely disseminated public health approach for identifying and addressing problematic alcohol and substance use within primary and other healthcare settings [1, 3, 4]. While evidence supporting SBIRT’s effectiveness is largely limited to improving outcomes for those with alcohol use disorder [5–8], a separate line of research focusing specifically on BT demonstrates it can be an effective, shorter-term, and lower-cost intervention for patients who use other substances [9, 10, 11]. However, inconsistent implementation of BT has been noted to result in treatment that looks similar to more intensive outpatient care [12, 13], thus blurring the lines between these two treatment types and making it difficult to attribute patient-level outcomes to BT received by patients. Despite this, prior research has not explicitly documented variations in BT practice. The current study addresses this gap through a qualitative investigation of the delivery of BT as part of an SBIRT model among four Federally Qualified Health Centers (FQHCs), as well as considering how BT delivery might impact perceptions of care among patients.

As a primary champion of the SBIRT model, the US Substance Abuse and Mental Health Services Administration (SAMHSA) has provided guidance regarding the modality, content, and duration of BT sessions [2]:

[BT] is a systematic, focused process that relies on assessment, patient engagement, and implementation of change strategies. The goal of BT is to change not only the immediate behavior or thoughts about risky behavior but also to address long standing problems with harmful drinking and drug misuse and help patients with higher levels of disorder obtain more intensive care. The treatment consists of assessment and a limited number (typically 6–20) of evidence-based, highly focused, and structured clinical sessions…to help patients address unhealthy cognitions and behaviors associated with current use patterns and adopt change strategies. ([2], p. 9)

While this statement provides guidance regarding the modality, content, and duration of sessions, prior research measuring the receipt of BT among SBIRT recipients has employed less comprehensive and consistent definitions. For instance, while most prior studies provide some reference to the optimal number of sessions, they range from 1 to 6 expected minimum and 5–8 expected maximum sessions [14–22]. Some studies have also defined the expected length of each session, with minimums ranging from 5 to 50 min and maximums from 60 to 120 min [14, 15, 18, 23]. Other studies have provided more compressive definitions that include expected content (e.g., motivational enhancement, motivational interviewing, cognitive behavioral techniques/therapy) or goals (e.g., develop skills, change behavior, educating, motivating, and reducing risky behavior) in addition to session number and length [17–19, 21, 22, 24]. Yet, other research has provided no clear example of how BT was operationalized or simply accepted that it was delivered when reported so by service providers [4, 25–31]. This inconsistent operationalization combined with a noted lack of fidelity measurement [32] in prior BT research raise validity and reliability concerns that hamper the quality of evidence on the intervention’s efficacy.

## Motivation for the current study

The motivation for conducting the current qualitative investigation of BT stems from an ongoing randomized clinical trial that is comparing SBIRT only with SBIRT + Recovery Management Checkups (RMC) among patients at four FQHCs [33, 34]. RMC is an evidence-based practice used to improve substance use treatment initiation, engagement, and retention. As part of the current trial, participating FQHCs conduct universal SBIRT screening using validated instruments. Patients who screen positive, qualify for referral to BT or regular treatment. Those who agree to participate are randomly assigned to a referral as usual group or to the RMC condition.

Since initiation and amount of treatment received are key outcomes in this trial, we conducted preliminary analyses to examine the initial impact of RMC. Of the first 15 RMC cases referred to and admitted to BT based on linkage manager records, 8 (53 %) self-reported no substance use treatment of any kind in the past 90 days. This is about 10 times higher than the rate for not reporting other kinds of SUD treatment, which raised concerns as to why patients failed to report having received BT.

Given there are no negative or positive consequences associated with reporting receipt of BT, we explored the source of the discrepancy from three angles. First, after speaking with research interviewers and treatment linkage managers, it was apparent that patients might not identify or label BT delivered in the context of an FQHC as “substance use treatment”. Correspondingly, when the interviewer asks the patient about treatment utilization in the past 90 days, the patient may not consider BT as “real treatment.” To help with this, our second step was to add a question to the research interview that specifically asks and provides an explanation about BT. Of the next 18 patients referred and admitted to BT based on records, 9 (50 %) reported no substance use treatment of any kind in the next 90 days. Of the 9 that reported some kind of treatment, 8 reported some days of BT based on the new BT specific question. Though a slight improvement, this solution failed to address the observed discrepancy.

Our third and final step was to look into the literature on SBIRT and BT. However, this failed to explain the discrepancies observed in the trial. Furthermore, as noted above, it established there were wide-ranging inconsistencies regarding BT descriptions in the SBIRT literature and that research and practice stand to benefit from greater agreement in this regard. As such, the current study aimed to understand the extent to which inconsistencies in real-world BT practice might exist by answering the following questions as they related to the RMC trial: (1) How is BT defined and structured within the participating FQHCs?; (2) Which patients are considered most appropriate for BT services?; (3) How do the FQHCs ensure quality of BT service delivery?; (4) Why do providers think some clinical trial participants did not recognize having received BT? To address these questions, we interviewed administrators and staff at the participating FQHCs.

## Methods

 Qualitative Interview participants were employees of the four FQHCs participating in the trial. We emailed the primary administrative contact at each FQHC requesting to conduct interviews with 3–5 staff and one administrator/manager who could speak knowledgably about BT within their organization. Of 13 individuals identified, we were able to complete interviews with two staff and one administrator (12 interviews total) from each FQHC.

The Chestnut Health System’s Institutional Review Board reviewed and approved all human subjects procedures. The first and second authors conducted and recorded individual semi-structured interviews over teleconference technology. Interviewers obtained verbal consent prior to the start of each interview, offering all interviewees a $25 Visa gift card as an incentive. The interview guide was structured using the primary research questions as a guide. As is standard practice in interpretive qualitative research, questions expanded beyond the original guide both within and across interviews as new information about the phenomenon of interest was learned [35]; however, core questions were retained throughout the course of data collection. The average interview time was around 30 min.

The first and second authors conducted the data analysis in MAXQDA 20.1 qualitative data analysis software [36]. Coding for all transcripts was completed after data collection concluded. The lead author first coded all transcripts following an inductive analysis approach whereby he developed an initial list of codes based on the four guiding research questions and then inductively developed subcodes within them [37]. Next, the second author independently reviewed the transcripts and lead author’s coding to identify areas of disagreement and coded passages of text that were overlooked. The two researchers then discussed disagreements and adjusted the coding appropriately until 100 % agreement was met. They then used MAXQDA’s quote matrix feature to compare codes/themes across the FQHCs to identify similarities and differences among them. The small recruitment pool limited the ability to add additional research interviewees to the sample. Therefore, the two analysts defined theoretical saturation at the point when incremental learning about the phenomenon of interest was no longer improved through iteration between developing themes and the data [38, 39]. As a last step, initial findings were discussed with the other three authors (who were more familiar with the larger clinical trial) to ensure findings fit with their broad understandings of the FQHC’s and how they functioned.

## Results

Below, we present the findings as they apply to each of the four research questions that guided data collection and analysis activities.

### Question 1: How is BT defined and structured within the participating FQHCs?

***How BT was defined.*** At least one interviewee (n = 7) at each FQHC *defined BT by its relative intensity* in comparison to other forms of SUD care. One interviewee compared BT to brief intervention, stating BT was “more formal and can be an ongoing process”. Others compared BT to higher levels of care. For instance, one stated that higher-level services involve a larger team of professionals, including “a physician and a therapist, and substance use counselor” working together to address a patient’s substance use issues, while BT usually involves only a single behavioral health provider. Two other interviewees defined BT as requiring engagement in fewer different types of treatment, while higher levels of care involve “more interventions that are happening at the same time” such as individual and group counseling. Finally, two interviewees discussed how higher levels of care require more frequent visits than BT: “So, if it’s [intensive outpatient], it’s probably four days a week. If it’s [outpatient], it could be 2 days a week. The difference with brief treatment…[is that] you do once a week, a 20–30-minute session.”

Interviewees (n = 5) from three of the FQHCs discussed how the *content of BT is what defines it* (i.e., what is discussed and/or what activities are done). Most of these discussions focused on early meetings and the importance of gathering information about the patient in an effort to determine needs, goals, and readiness for making behavioral changes: “…it [BT] is kind of breaking down where they at in their phase of change…And then, you kind of going through what are their goals”. While activities such as this are conducted in other levels of treatment, these discussions demonstrated how some interviewees understood the primary difference to be that BT patients are often not open to discussing their substance use. Because of this, clinicians need to find topics patients are willing to discuss to keep them engaged and then use that as a motivating leverage. One discrepancy related to this theme is that one interviewee noted “in brief treatment, we really don’t do goals. Because once again, it’s brief. They don’t have time to identify and work on goals”. This interviewee then went on to state that goal setting was appropriate for more intensive treatment.

At one FQHC, two interviewees described the defining feature of BT in their clinic as its *integration with other healthcare services*. While specific appointments for BT could be scheduled, staff stated sessions tended to occur when the patient was visiting the clinic for services related to their physical health: “It [BT] is not like, ‘hey, you’re going to be in treatment’. It’s more, ‘would you be open to meeting with me and having another conversation in a month when you meet, when you happen to be here for your doctor’s appointment’”. The reasons explained for this is that most BT patients were not open to coming in for separate visits related to their substance use and received all of their behavioral health services in the main clinic area as a result.

***How BT sessions are structured.*** BT sessions were structured by the number and length of sessions patients received and the activities of which they could be composed. There were substantial differences between both the *expected number and duration of BT sessions* among the FQHCs. Regarding session number, two FQHCs did not have any expected minimum or maximum established, another ranged from 5 to 9 sessions, and the fourth ranged from 3 to 6 sessions. Furthermore, interviewees at 3 of the FQHCs stated they would keep working with a patient as long as they continued to benefit from BT. Regarding session length, one FQHC’s sessions were slated for 30 min but staff might accommodate to provide up to an hour if their schedule allowed. A second FQHC had two different BT tracks, one lasting 20 min and the other 50 min. The last two FQHCs had defined time ranges of 20–40 min and 16–30 min respectively.

We identified 9 activities that interviewees discussed when *describing the content of BT* sessions. Only two of these activities were discussed by interviewees (n = 6) at all four FQHCs. The first was *motivational interviewing.* As the quote below demonstrates, motivational interviewing is a technique that allows the clinician to focus on topics important to the patient (e.g., depression, smoking cessation, weight loss, physical health, family, housing), while using specific conversational strategies to help them understand how these issues might relate to their substance use and engage them in a process of change [40]:

That’s where motivational interviewing comes in. You know, we’re not forcing people, we’re making recommendations and talk about health issues and what they want to accomplish. [If a patient says]“oh I want to feel, I’m tired of feeling depressed”. [A response would be] “Okay, well you say that, but [do] you think not drinking would be helpful? Because we know that drinking is a depressant, so it’s going to add to the depression.”

The importance of motivational interviewing was underscored by sentiments that BT patients often have not yet recognized their substance use as problematic or they are not yet ready to take action necessary to address risky substance use behaviors.

The other activity discussed by interviewees at all four FQHCs was *planning*, and it was discussed in two ways. The first type of planning discussed (n = 3) was related to development of the treatment plan, which entails identifying the “avenue that they [the patient] want to go in” and “developing more concrete steps” to help get them there. The second type of planning discussed (n = 5) was relapse prevention, which involves identifying the patient’s prior use patterns and triggers and how they will use coping skills and recovery supports to prevent a relapse from occurring: “[In BT] we do a lot of relapse prevention, we do a lot of coping skills”.

The other 8 activities were discussed by interviewees at three or fewer FQHCs, and they include: screening/assessing, checking in on quality of life, building rapport, educating, skill building, goal setting, and connecting to external resources/services. As they were discussed less than the prior two themes, we have provided a description of each activity with a definition and example quote in Table 1.

**Table 1:** Activities described in interviews as composing the content of brief treatment (BT) sessions

Activity	# of FQHCs where activity was discussed	# of interviewees discussing activity	Thematic definition	Example quote
Motivational interviewing	4	6	Initiating client-centered discussions to help patients identify connections between personal areas of concern and their substance use.	I do a lot of the motivational interviewing, so it is really kind of breaking down where are they at in their phase of change.
Planning	4	6	Developing concrete plans to guide treatment and/or relapse prevention strategies.	…that would be the topic, you know, substance abuse, you know, going to meetings, relapse prevention skills, utilizing, you know, your recovery support. Developing a relapse prevention plan.
Screen/Assess	3	3	Collecting information in early BT sessions to understand the patient’s current substance use, mental health, physical health, and/or social situations.	…in the first session…I’m going to do the initial depression, anxiety, SBIRT, all those screeners.
Checking in on quality of life	3	6	Getting updates regarding various areas of patients’ lives (e.g., mental/emotional, health, physical health, substance use, family).	…then we’re going to go through areas of rating their individual components of their life: mood, diet, exercise, sleep, pain.
Rapport building	2	2	Establishing a relationship with patients to improve their comfort levels with the treatment being provided and/or changing their behaviors related to substance use.	And you know, [you] want them to open up, and so maybe if we’re kind of noticing that there is push back, [I] try not to focus too much on how much they use or what they’re using.
Educate	2	2	Providing information to patients.	I’d always say, you know, can I provide you some education, or can I provide some information on that.
Skill building	2	3	Building coping/relapse prevention skills either in sessions or through homework.	[We] talk about that and work on “what are your coping skills, what are your mechanisms”.
Goal setting	2	3	Identifying areas of their life that patients want to improve.	…going through, what are their goals. So, if their goals are to decrease use, we can focus on that….
Connect to external resources/ services	2	3	Making patients aware of or providing a direct referral to additional resources or services outside of BT.	If they need more residential [services] or they need more intensive [services]…a lot of time our services are not enough for someone who is trying to maintain their sobriety.

### Question 2: Which patients are appropriate for BT services?

There were discussions with interviewees from all four FQHCs (n = 6) demonstrating how *patients with less severe substance use issues*—as determined by either SBIRT screening or their presenting symptoms—were generally appropriate candidates for BT. Additionally, those with more severe issues were framed as being more appropriate for referral to a higher level of treatment: “…when it comes to someone who is still having a lot of, they’re currently using or they’re still trying, still having a lot of cravings and triggers and things like that, they need more of an intensive treatment.”

*Patient interest* was another qualifier for BT discussed by participants (n = 7) at every FQHC. These discussions generally demonstrated the SBIRT process would lead to an initial recommendation for BT or referral to a higher level of treatment, but it was ultimately up to the patient to determine what level of care they were comfortable with. At one FQHC, they even changed procedures to ensure interest in BT by requiring patients to call and schedule their first appointment: “…so, at first they [staff at the referral agency] would schedule an appointment for the [patient]. And, the change was for the [patient] to schedule the appointment for themselves because we felt like that gave us some idea that the [patient] was more interested….”.

Interviewees (n = 2) at two FQHCs stated that a patient’s *issues needed to be addressable within a small number of focused sessions* to be appropriate for BT. For instance, one interviewee discussed how patients whose substance use might be driven by recent grief could be more appropriate for BT because they “….may be almost there at the acceptance stage [accepting the loss of a loved one], right? And, she may not need a whole lot of on-going individual sessions”.

### Question 3: How do the FQHCs ensure quality of BT services delivered?

*None of the interviewees could directly identify any procedures* implemented at their FQHC to ensure or assess quality of BT services. Furthermore, outside what was provided as part of the RMC-PC clinical trial, none of the FQHCs provided any training in BT to staff. They instead relied largely on prior clinical training to have prepared staff to deliver BT: “So, if somebody is coming from another type of clinic or healthcare setting or [has a] substance use background…so those people are going to be much more likely, they probably know what it [BT] is.” For these reasons, BT within the FQHCs likely varied considerably by clinician as a function of their prior training and experience.

A final aspect of BT service quality investigated was related to *referral and follow-up procedures*. Only interviews from two FQHCs described a strong process for BT referral tracking that included both (a) tracking if patients made their BT appointments and (b) some method of following up with those patients who did not show for appointments: “…that [tracking and following up] is the policy here. Yes, we make usually around 2 or 3 attempts [to contact them if they miss an appointment]”. One FQHC had a general agency-wide tracking process, but discussions demonstrated interviewees were unfamiliar with it and that it was also not used consistently with BT patients. Finally, one FQHC tracked whether patients showed to their appointments, but did not follow-up with new patients who missed them because staff “don’t have time to do so”.

### Question 4: Why do providers think some trial participants did not recognize having received BT?

There were four categories of reasons attributed to the inability of some RMC-PC clinical trial participants’ to recognize that they had received BT. Interviewees (n = 5) at all four FQHCs discussed how BT patients often *do not recognize they have a substance use issue* and do not attribute their interactions with staff to be related to SUD treatment. More than one interviewee discussed how patients in earlier “stages of change” are likely not ready to admit they have a substance use issue:

Maybe they’re [patients who do not recognize BT received] not really looking at it as treatment, cause like I said, a lot of time when we’re getting that referral, the client isn’t actually at that point in being ready to change…they might not even be aware that they’re receiving treatment.

Despite this common sentiment, none of the FQHCs had an objective way of measuring the stage of change a patient was in or reported using a standardized assessment to do so. As such, this determination was based on the judgment of the clinician providing the treatment.

A second category of explanations discussed by interviewees (n = 9) at all the FQHCs was that patients might not recognize having received BT because it is *not explicitly framed as SUD treatment*. These respondents felt patients’ understanding was influenced by “how the clinician explains it [BT]”, and most clinicians at the FQHCs do not actually use the terms “brief treatment” or “substance use disorder treatment” when describing BT services to their patients. Indeed, only one FQHC actually used the term “brief treatment” in staff interactions, but the term “session” was generally used to describe BT services to patients. The other FQHCs used the terms “sessions’, “individual therapy”, and “integrated care” when referring to BT among both colleagues and patients.

Interviewees (n = 5) at three of the FQHCs discussed how treatment *might not be structured or focused in a way that the patient is able to recognize it as any form of SUD treatment*. Two of these interviewees discussed how BT was often integrated with other types of care, so patients are more likely to recognize it as part of a regular physical healthcare or another type of service visit:

And because they were receiving other services there [in the main clinic], I would just kind of snatch them, pull them in to my office and do a brief treatment…but to them that may not have been looked as a brief treatment because they were actually there to see the doctor that day for medical [reasons].

Another way this was discussed was how the focus of BT is often on other issues besides substance use. This is because the patient is often viewed as not being in a space where they are ready or willing to address the issue or there are other factors underlying their use that need to be addressed: “a lot of times when we do have brief treatment, our sole focus is not so much on the use of alcohol or drugs. We kind of explore other things that maybe lead up to those things, past traumas and things like that”. The previous two quotes also demonstrate how BT often does not look like what patients expect SUD treatment to be based on their previous experiences or notions that are often structured around abstinence as a goal: “[if] the interaction with the counselor or clinician isn’t towards abstinence, then maybe that’s confusing for people who are engaging in it [BT]. Maybe it does not feel like traditional substance use treatment”.

Finally, a single interviewee from an FQHC with no limits on BT sessions pointed out how patients might not know they received BT when asked because *it can extend over an indefinite amount of time*: “They [the patient] may not [recognize it as BT], you know, ’oh, I’ve been seeing [name of staff person who provides BT], I saw her six times, no that’s not brief’”. This discussion also demonstrated those receiving BT might be long-term patients of the FQHC who do not necessarily differentiate BT from the other services they receive there.

## Discussion

There are considerable differences in how BT is conceptualized and implemented within the FQHCs participating in the RMC-PC clinical trial. As previously noted, the SBIRT literature is rife with conflicting and incomplete descriptions of BT, including studies conducted in FQHC settings similar to those of the current sample [22, 27]. While prior-employed operational definitions of BT provide internal validity for individual studies, their wide variation brings into question the comparability of results attributed to SBIRT models with high levels of BT referral. Additionally, this variation highlights the need for a more standardized and comprehensive operational definition that goes beyond number and length of treatment sessions. Our findings also raise internal validity concerns for any studies that have relied on provider reports or administrative data to measure BT received [19, 25, 30, 41–43], particularly when said studies work with more than one providing organization. Variability in real-world BT practice also brings into question the external validity of studies, as BT as operationalized in research is likely different from what patients encounter in actual practice [44].

SAMHSA’s BT description discussed at the beginning of this paper provides a reasonable standard from which to compare our findings given the agency’s considerable work promoting and supporting implementation of the entire SBIRT model [2], and our findings have both compatibilities and incompatibilities with SAMHSA’s description. For instance, SAMHSA’s use of the term “limited intensity” is compatible with interviewee descriptions of BT as being less intensive than higher treatment levels, as well as those discussions demonstrating expected limits on the number and length of BT sessions. In addition, much of SAMHSA’s definition focuses on implementing change strategies as well as preparing the patient to enter a higher level of treatment. This is compatible with interviewee discussions of BT content as being focused on patient needs and readiness to make changes in their lives and the use of motivational interviewing in BT sessions. However, SAMHSA provides a guideline of 6–20 sessions, while 3 FQHCs in our sample continue working with patients as long as clinicians deem necessary. SAMHSA also defines BT sessions as being highly focused and structured, and this was both supported and contradicted in our findings. While discussions pointing to patients needing to have “addressable issues” and sessions as being goal focused is compatible with SAMSHA’s description of BT as a “systematic, focused process”, there was one interviewee who noted that BT sessions were too short to focus on goals. Our findings also point to a number of activities interviewees discussed carrying out as part of BT that do not contradict but are not referred to in SAMHSA’s definition (e.g., screening/assessing, checking in on quality of life, building rapport, educating, skill building, goal setting, connecting to external resources/services, and following up). The lack of explicit mention of these activities by SAMHSA could be because they overlap highly with the goals and tenants of motivational interviewing [40]; though, it is questionable as to why interviewees discussed these activities separately. Finally, at no point did interview participants mention the need to employ evidence-based approaches in BT, and this is a particular concern considering issues with evidence-based strategy implementation that have been noted across the SUD treatment field and with SBIRT specifically [45–48].

The findings also point to an apparent lack of training and quality assurance standards for BT within the participating FQHCs. Lack of strong tracking and follow-up procedures are particularly concerning considering referral activities are a key aspect of SBIRT coordination [15, 49]. Indeed, a recent systematic review noted that prior SBIRT studies have been limited in their description of the referral process [7], pointing to potentially fruitful area for future investigation. Additionally, reliance on clinical judgment rooted in staff members’ prior training and experience suggests a lack of standardization across systems and within individual care settings despite the availability of several standardized protocols and assessments of motivation and readiness [50, 51]. Given that higher-fidelity implementation and quality assurance procedures have been demonstrated to mediate client outcomes for behavioral health interventions [52, 53], another potential direction for future research in this area would be to test implementation strategies and quality assurance procedures and measure their effect on BT practice and associated patient outcomes (see [54]).

The patients considered to be the best candidates for BT where those who had addressable issues or who were ready to address their substance use. However, it was also pointed out that most BT patients are not ready to admit they have an issue or to take action regarding it. This is a contradiction that future research could explore to better understand BT referral decisions. For instance, it is possible patients who might not be considered the best candidates are nevertheless referred to BT services as a strategy for overcoming stigma that is a noted barrier to SUD treatment access [1, 55]. Many of these same issues underlie reasons offered as to why some patients might not recognize having received BT. This raises the question as to whether it even matters if patients are explicitly informed that they are receiving SUD treatment or whether it might even have negative connotations for patients who do not perceive themselves as having a substance use problem. Research investigating patient readiness/stage of change as a moderator of treatment outcomes could be useful in answering questions related to this issue [51, 56].

While the interviews provided rich information regarding BT practice, the small sample size and the FQHC’s participation in the same clinical trial might have influenced BT delivery, and thus limit generalizability of the findings. However, variations observed do align with documented inconsistencies in BT definition and implementation previously reported in the literature discussed above, and this supports the assertion that issues raised extend beyond those identified our data. A broader survey of BT as defined and practiced among a larger and more representative sample could aid in identifying best BT practices and the degree to which BT in clinical practice is aligned with BT as tested in research. This is the type of information that could prove useful for developing and testing more refined and potentially more effective models of BT approaches. The findings related to why BT patients might not identify having received treatment is also limited in that it reflects the opinions of clinicians, and future research in this area should seek to directly understand patient experiences and perceptions.

## Conclusions

This qualitative investigation identified considerable BT variations among four FQHCs involved in a clinical trial of RMCs, as well as identifying potential explanations as to why some patients who received BT during the trial did not recognize having had any form of SUD treatment. Such findings call into question validity of prior research in this area that rely on patient reports, provider reports, or administrative data to confirm study participants’ BT uptake and engagement. Stronger and more objective definitions of BT and fidelity checklists can help ensure internal validity and better comparison of results across different studies. Future research in this area should seek to understand BT as practiced among a larger sample of different provider types, as well as patients’ direct experiences and perspectives. Regardless of research needs, the BT practice variations and relatively weak internal standards identified lend support issues raised in prior research [12, 13, 32], thus reinforcing the need for more consistent implementation and quality assurance guidelines in FQHCs and other settings where BT is implemented. Finally, the importance of a client’s stage of change for determining BT appropriateness and content suggests the use of standardized assessments could be employed to guide clinical decision making in this area (see [51]).

## Data Availability

Qualitative data are not available due to confidentiality concerns related to such a small sample.

## Abbreviations

BT: Brief treatment
FQHC: Federally Qualified Health Center
SBIRT: Screening, Brief Intervention, and Referral to Treatment
SAMHSA: Substance Abuse and Mental Health Services Administration
SUD: Substance use disorder
